# A Direct Cortico-Nigral Pathway as Revealed by Constrained Spherical Deconvolution Tractography in Humans

**DOI:** 10.3389/fnhum.2016.00374

**Published:** 2016-07-26

**Authors:** Alberto Cacciola, Demetrio Milardi, Giuseppe P. Anastasi, Gianpaolo A. Basile, Pietro Ciolli, Mariangela Irrera, Giuseppina Cutroneo, Daniele Bruschetta, Giuseppina Rizzo, Stefania Mondello, Placido Bramanti, Angelo Quartarone

**Affiliations:** ^1^Department of Biomedical, Dental Sciences and Morphological and Functional Images, University of MessinaMessina, Italy; ^2^IRCCS Centro Neurolesi “Bonino Pulejo”Messina, Italy

**Keywords:** substantia nigra, CSD, basal ganglia, tractography, connectivity, cortex, parkinson disease

## Abstract

Substantia nigra is an important neuronal structure, located in the ventral midbrain, that exerts a regulatory function within the basal ganglia circuitry through the nigro-striatal pathway. Although its subcortical connections are relatively well-known in human brain, little is known about its cortical connections. The existence of a direct cortico-nigral pathway has been demonstrated in rodents and primates but only hypothesized in humans. In this study, we aimed at evaluating cortical connections of substantia nigra *in vivo* in human brain by using probabilistic constrained spherical deconvolution (CSD) tractography on magnetic resonance diffusion weighted imaging data. We found that substantia nigra is connected with cerebral cortex as a whole, with the most representative connections involving prefrontal cortex, precentral and postcentral gyri and superior parietal lobule. These results may be relevant for the comprehension of the pathophysiology of several neurological disorders involving substantia nigra, such as parkinson's disease, schizophrenia, and pathological addictions.

## Introduction

Substantia Nigra (SN) is a neuronal structure located in the ventral part of the midbrain, between crus cerebri and tegmentum. The postero-medial region of SN, known as “pars compacta” (SNc), is one of the four primary dopaminergic nuclei of the brain, together with the ventral tegmental area (VTA), the retrorubral area and the arcuate nucleus of hypothalamus (Nestler et al., 2009), and it is mainly connected with the dorsal striatum through the nigro-striatal pathway (Voorn et al., 2004). The antero-lateral zone, the “pars reticulata” (SNr), consists of GABAergic neurons which receive afferents from the striatum and subthalamic nucleus (STN) and in turn project to ventral-anterior (VA) and ventral-lateral (VL) thalamic nuclei (Zhou and Lee, 2011).

It is well-known that SN exerts a regulatory function on the basal ganglia circuitry (Guatteo et al., 2009), and it is involved in several neurological and neuropsychiatric disorders, such as Parkinson's Disease (Carman, 1968), schizophrenia (Weinberger, 1987), and pathological addictions (Wise, 2009).

Although several reports have demonstrated the existence of an extensive sub-cortical network for both SNc and SNr (Düzel et al., 2009; Menke et al., 2010; Chowdhury et al., 2013), less is known about the existence of direct cortico-nigral connections in the human brain.

The existence of a direct cortico-nigral pathway was postulated for the first time by Foix and Niculescu (1925) and then reproposed by Rinvik and co-workers, who suggested the presence of direct projections arising from premotor area and reaching SN (Rinvik, 1966).

However, it was only the 1974 when direct cortico-nigral connectivity was demonstrated in cats by means of fiber degeneration methods (Fink-Heimer staining) (Afifi et al., 1974). On the contrary some studies in primates failed to reveal representative direct connections between cortex and ventral midbrain, resulting only in sparse or isolated fiber tracts (Leichnetz and Astruc, 1976; Künzle, 1978; von Monakow et al., 1979).

Further investigations in rodents revealed cortico-nigral connections between prefrontal cortex (PFC) and SN, using antero- and retrograde fiber tracers (Bunney and Aghajanian, 1976; Beckstead, 1979; Sesack et al., 1989; Naito and Kita, 1994; Jasmin et al., 2004; Zakiewicz et al., 2014; Höglinger et al., 2015) and ablation techniques (Carter, 1982; Kornhuber, 1984).

In addition, in an auto radiographic tracing study, it was found that area 6 in the raccoon projected to the ipsilateral SNc (Sakai, 1978).

Finally, in a more recent study on macaca monkeys, Frankle et al. (2006) injected anterograde tracers into the orbital (OFC), cingulate and dorsolateral prefrontal (dlPFC) cortices, demonstrating direct connections from OFC and dlPFC to SN, whilst cingulate cortex was mainly connected to VTA (Frankle et al., 2006).

On the one hand, even if tract-tracing methods are the gold standard for studying the anatomo-physiology of basal ganglia, on the other hand, they still remain highly invasive techniques and therefore they cannot be used to study human brain *in vivo* (McFarland and Haber, 2000; Nambu et al., 2000; Kita, 2001).

However, in recent years, a growing interest in neuroimaging studies and several developments in magnetic resonance imaging have permitted to shed new light on the whole brain connectivity.

In this regard, Diffusion Tensor Imaging (DTI) is a modeling technique which allows, from the analysis of anisotropic water motion in white matter, the non-invasive reconstruction, and visualization of white matter fiber bundles (Mormina et al., 2015); it thus permits to estimate connectivity patterns between distinct brain regions (Basser et al., 1994; Henderson, 2012; Le Bihan and Johansen-Berg, 2012). Tractographic findings strongly depend on the diffusion model adopted: indeed, it is known that DTI suffers from several limitations such as large reconstruction biases and less reliability for fibers with complex configuration (e.g., crossing, fanning, kinking fibers; Parker and Alexander, 2005; Behrens et al., 2007; Jones and Cercignani, 2010; Farquharson et al., 2013). Consequently, several sequences and related signal modeling have been recently developed and used for exploring neural connectivity in normal subjects (Parker and Alexander, 2005; Jbabdi and Johansen-Berg, 2011; Farquharson et al., 2013; Jang et al., 2013; Jbabdi et al., 2015).

In particular, constrained spherical deconvolution (CSD) is able to reduce reconstruction biases and to provide more robust data, estimating one or more fiber orientations in presence of intravoxel orientational heterogeneity (Tournier et al., 2007, 2008; Farquharson et al., 2013; Milardi et al., 2016b).

To date, only few multitensorial DTI studies have focused on the connectivity of SN in human subjects (Menke et al., 2010; Chowdhury et al., 2013). In particular, Menke et al. used probabilistic DTI tractography with the aim of differentiating SNc and SNr according to their different connectivity profile. They demonstrated that these regions are both likely connected to cerebral cortex by means of the thalamus. SNc showed higher connectivity profile with prefrontal cortex, whilst SNr was more connected with motor and premotor cortices (Menke et al., 2010). Chowdury et al. investigated the well-characterized anatomical projections between the SN/VTA and striatum, providing a new method for subdividing the SN/VTA based on connectivity to the striatum (Chowdhury et al., 2013). Furthermore, they claimed that “direct projections between the SN/VTA and other structures exist, for example with the prefrontal cortex (Künzle, 1978).”

Finally, Kwon and Jang (2014) directly aimed the question of different patterns of connectivity between SN/VTA, showing that SN is connected with several brain structures such as corpus callosum, primary sensory cortex, premotor cortex, caudate nucleus, putamen, nucleus accumbens, temporal-occipital lobes, pontine basis, anterior lobe of cerebellum, external capsule (Kwon and Jang, 2014).

Challenging the classical view of the basal ganglia network based on the “direct,” “indirect” (Leblois et al., 2006), and “hyperdirect” pathways (Nambu et al., 1996, 2002), we have recently demonstrated the existence of a possible cortico-pallidal connectivity in humans (Milardi et al., 2015; Smith and Wichmann, 2015), which was then corroborated by a magnetoencephalography-local field potentials study in dystonic patients (Neumann et al., 2015). Furthermore, this hypothesis would meet the vision that the cortico-basal ganglia network consists of several, parallel, segregated, and functionally distinct, but homologous loop.

In this framework, we supported the hypothesis that the SN is not only a part of the subcortical basal ganglia network, but it is also connected with cortex, by means of an additional, parallel circuit. In the present study we provided tractographic findings of the existence of a cortico-nigral pathway in humans, confirming the previously existing literature based on animal studies.

## Materials and methods

### Participants

We recruited a total of 15 human subjects (male: 8, female: 7; average age 35.46 ± 11 years) with no previous history any overt neurological, physical or psychiatric disease. The research followed the tenets of the Declaration of Helsinki; written informed consent was signed from all included subjects, after explanation of the nature and possible consequences of the procedure. The study was approved by the institutional review board of IRCCS Bonino Pulejo, Messina, Italy (Scientific Institute for Research, Hospitalization and Health Care), protocol number 15/2012.

### Data acquisition

The study was performed with a 3T Achieva Philips scanner using a 32-channels SENSE head coil. In each patient a structural 3D high-resolution T1 weighted Fast Field Echo (FFE) sequence was acquired using the following parameters: repetition time 25 ms; echo time 4.6 ms; flip angle 30°; FOV 240 × 240 mm^2^; reconstruction matrix 240 × 240; voxel size 1 × 1 × 1 mm; slice thickness 1 mm. The acquisition time was 6 min. Furthermore, a 3-D high resolution T2 weighted Turbo Spin Echo (TSE) sequence was obtained using the following parameters: repetition time 2500 ms; echo time 380 ms; FOV 250 × 250 mm^2^; reconstruction matrix 312 × 312; voxel size 0.8 × 0.8 × 0.8 mm; slice thickness 0.8 mm, The acquisition time was 9 min and 38 s.

**Figure 1:**
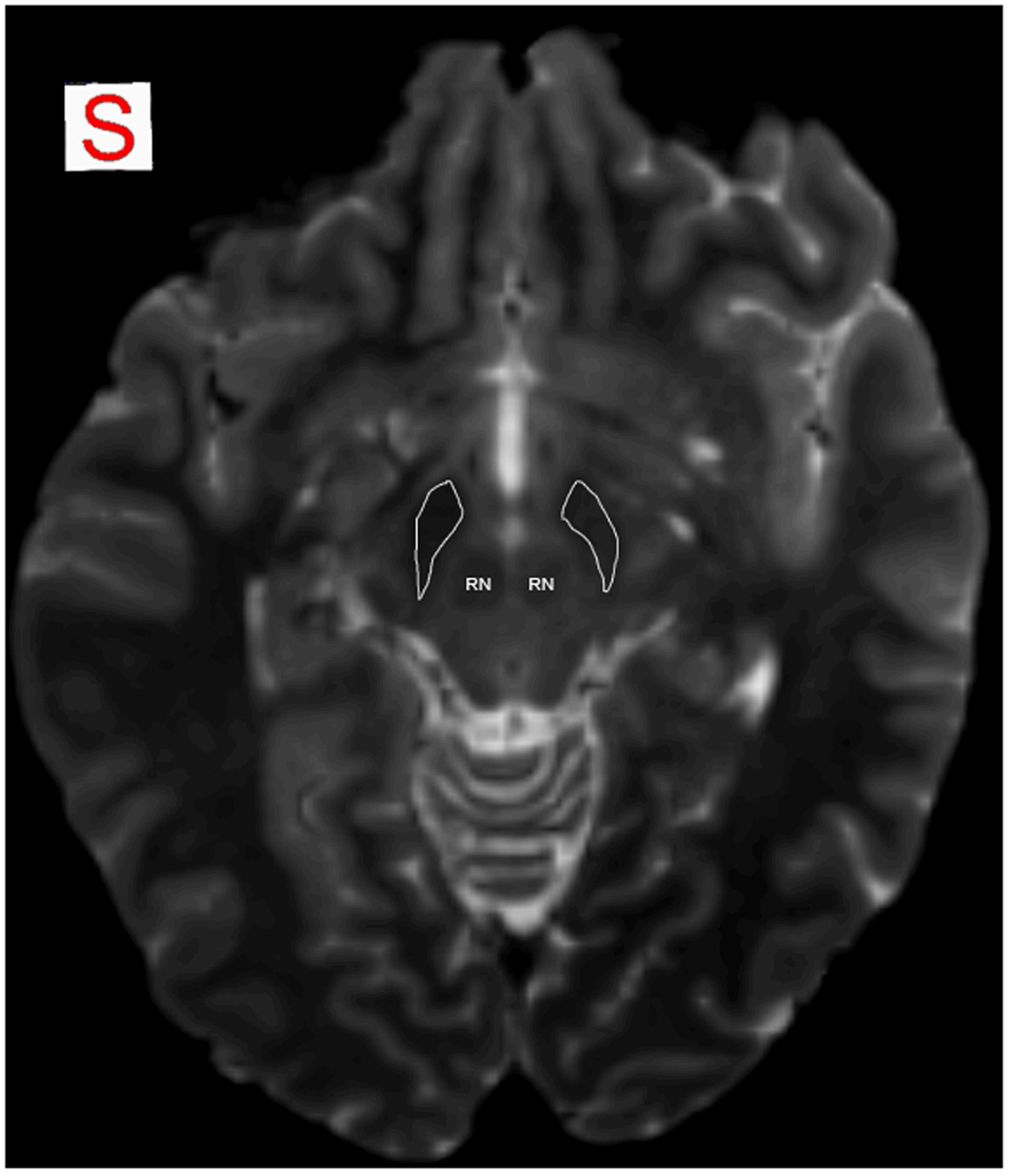
**MRI identification of SN**. SN is detectable as an hypointense region in axial plane in T2-weighted images, due to T2^*^ effect that allows a better visualization of the iron-loaded nuclei. Anatomical relations of SN with RN are well identifiable. RN, red nucleus; SN is encircled in white.

The use of 3D TSE sequence allowed obtaining high-resolution images with a relative short acquisition time. At same time this sequence permitted to obtain a fine representation of the iron loaded nuclei due to T2^*^ effect linked with the use of a very long echo-time (Figure 1).

Furthermore, a DWI dataset was obtained with a dual phase encoded pulsed gradient spin echo sequence (Embleton et al., 2010); following parameters were adopted: *b*-value 1500 s/mm^2^, 61 gradient diffusion directions evenly distributed on a sphere more 3 un-weighted b0 volumes, echo-time 54 ms, repetition time 11,884 ms, FOV 240 × 240 mm^2^ resulting in isotropic 2 mm voxel resolution.

### DWI pre-processing and co-registration

All diffusion images were corrected for motion as well as for Eddy Currents distortion artifacts using tools available within SPM8 MATLAB tool (http://www.fil.ion.ucl.ac.uk/spm/software/spm8/) and FSL. In brief, affine transformation based on b0 volumes was estimated and applied to diffusion images using Diffusion tool (https://sourceforge.net/projects/spmtools/); a displacement field map was created using topup FSL function on the basis of two b0 images acquired in opposite phase encoding directions. Such displacement field map was eventually fed to diffusion dataset using Unwarp and Fieldmap SPM8 toolboxes (http://www.fil.ion.ucl.ac.uk/spm/toolbox/unwarp/). Rotational parts of transformations were applied to individual gradient directions. High Resolution T1 and T2 images were then co-registered to preprocessed DWIs using a pipeline outlined in (Besson et al., 2014): in particular, Cerebral Spinal Fluid (CSF) masks were estimated from average of b0 images as well as from T1 ones using New Segment SPM8 utility. Then, CSFs of structural scans were warped to match CSF estimated from b0-image using FLIRT and FNIRT FSL utilities (http://fsl.fmrib.ox.ac.uk/fsl/fslwiki/). Deformation fields were eventually applied to T1s to obtain an alignment as accurate as possible between structural and diffusion data.

This workflow led to much better cortical and subcortical correspondence than traditional affine co-registrations involving T1 and DWIs. In this way, we minimized possible misalignment biases coming from usage, in diffusion images space, of ROIs segmented in space of structural scans. This process is therefore fundamental to have an optimal anatomical correspondence, avoiding Regions of Interests (ROIs) to be slightly displaced and affected by CSF signal.

### Segmentation of SN

The use of a very long echo-time in 3D T2-weighted TSE sequences allowed to obtain a reliable identification and segmentation of the iron loaded nuclei. In particular, by means of a viewer provided together with MRtrix package, the mask for SN was manually outlined by one of the authors bilaterally in T2-weighted images in the axial plane (Figure 1) superimposed on the color-coded FA map, where the SN typically appeared in green (Mori et al., 2004; Kwon and Jang, 2014). We localized the SN immediately above the pons, as a hypointense region between crus cerebri and tegmentum. Ascending in caudo-cranial direction, this region appeared to be expanded ventrally and we localized behind it the red nucleus (RN), in a more medial position. We were able to identify VTA as the medial hyperintense zone between RN and SN. In the upper sections of SN, we identified the STN as dorso-medial boundary and marked the SN in every ascending slices until the superior colliculi and the cerebral aqueduct disappeared. As posterior and anterior boundaries of the most superior section, the cerebral aqueduct and mammillary bodies were, respectively, delineated. STN becomes prominent in the axial plane including third ventricle, RN and the posterior commissure. The mask was better defined in both sagittal and coronal planes. It is worthy to note that the mask of SN was outlined small enough to avoid other white matter voxels and fiber streamlines in a neighboring area.

### Cortical parcellation

Cortical reconstruction and volumetric segmentation were performed on co-registered T1 images with the Freesurfer image analysis suite, which is documented and freely available for download online (http://surfer.nmr.mgh.harvard.edu/). Briefly, this processing involves motion correction and averaging (Reuter et al., 2010) of T1 weighted images, removal of non-brain tissue using a hybrid watershed/surface deformation procedure (Segonne et al., 2004), segmentation of the subcortical white matter and deep gray matter volumetric structures (Fischl et al., 2004), tessellation of the gray matter white matter boundary, automated topology correction (Ségonne et al., 2007), and surface deformation following intensity gradients to optimally place the gray/white and gray/cerebrospinal fluid borders at the location where the greatest shift in intensity defines the transition to the other tissue class (Fischl and Dale, 2000). Once the cortical models were completed, parcellation of the cerebral cortex into units with respect to gyral and sulcal structure (Desikan et al., 2006) was performed. Successively, the obtained parcellation and segmentation of each individual were visually inspected and, if needed, manually edited by one of the authors.

### Tractography

To model diffusion signal, we used a modified High Angular Resolution Diffusion Imaging (HARDI) technique called non-negative CSD: this technique estimates the fiber Orientation Distribution Function (fODF) directly from deconvolution of DW signal with a reference single fiber response function (Tournier et al., 2007). Response function was estimated from the data on the basis of voxels having a Fractional Anisotropy index (FA) above 0.7, this threshold being a strong indicator that a single fiber population insists within those voxels. fODF estimation and tractography were performed using MRtrix software (http://jdtournier.github.io/mrtrix-0.2/index.html).

Using CSD to extract local fiber orientations we managed to overcome partial volume effects associated with DTI and also to improve, comparing with other types of HARDI acquisitions, the poor angular resolution achieved with QBI (Q-balls Imaging), while discarding DSI due to its longer acquisition time (Alexander and Barker, 2005; Tournier et al., 2008).

In our study, spherical harmonic degree was fixed equal to 8 in order to obtain robustness to noise. During tractographic reconstruction, tracking was stopped when one of the following conditions existed: step size = 0.2 mm, maximum angle = 10°, minimal fODF amplitude = 0.15. The latter parameter allowed to obtain more accurate reconstructions avoiding streamlines to enter GM in deep or passing through CSF; indeed, in those regions, estimated fODF amplitudes are lower than such cut-off. This is a more conservative choice with respect to usual standards, since we preferred to underestimate fiber bundles in order to have consistent reconstruction without false positive (Descoteaux et al., 2009; Tournier et al., 2011).

We performed, for each subject, probabilistic whole brain tractography by generating a total of one millions streamlines using WM masks both as seed and mask regions; before running tractography, we applied a small dilatation to WM masks in order to allow streamlines to reach our ROIs, placed in GM, for subsequent analyses.

A further improvement in the definition of the fiber tracking was obtained by an anatomical model-based approach using regions of avoidance (ROAs) that, on the contrary, “filter out” the tracks (Verstynen et al., 2011). In particular, based on whole brain tractography, we have filtered out tracks by using SN as inclusion mask and basal ganglia as exclusion ones, isolating white matter reconstructed bundles traveling from SN to cerebral cortex in the internal capsule, from other potential paths that do not travel in the internal capsule and/or passing through the basal ganglia (and as such would not be direct). We excluded thalamus, caudate, putamen, globus pallidum (GP), STN, and corpus callosum. Since the putamen and pallidus were used as ROAs, it was fundamental that they did not include part of the internal capsule; indeed, the mask of these two regions were manually drawn by keeping only voxels with FA values lower than 0.2. This step was performed to exclude from the analysis fibers passing through the basal ganglia (Grèzes et al., 2014).

For visualization purposes, we reconstructed a color-coded map in which red, blue, and green colors indicate the principal streamline directions (Pajevic and Pierpaoli, 1999). Specifically, red color indicates a latero-lateral pattern, green color an anterior-posterior pattern, and blue color a caudal-cranial pattern. Intensity and pureness of these colors vary according to the behavior of fiber bundles in all intermediate positions.

### Analysis of diffusion tensor parameters

We extracted for each pathway mean fractional anisotropy (FA) parameter defined by Basser (1995). Diffusion tensors were estimated with a non-linear procedure, which allowed to minimize unrealistic results (Jones and Basser, 2004), by means of CAMINO software package (http://cmic.cs.ucl.ac.uk/camino/). By using in-house scripts built with MATLAB software package (http://www.mathworks.com), we extracted FA for each pathway.

### Connectivity analysis

Once whole brain probabilistic tractography process terminated, for each subject we isolated streamlines linking SN with cortical areas previously parcellated on structural scans. Then, we calculated number of streamlines connecting each couple of ROIs. With some limitations (Smith et al., 2013), such numbers are used as markers of connectivity density, both in healthy and pathological conditions (Behrens and Sporns, 2012; Bijttebier et al., 2015; Li et al., 2016).

This approach is defective since streamlines are generated without considering the amount of white matter they represent and information such as the size of ROIs and the distance between ROIs.

Then we took into account for volume and distance biases, since it is documented that streamlines are more likely to connect with larger ROIs or closer ROIs. In this sense, we performed weighted connectivity defined as the density of the streamlines linking a couple of ROIs, which definitely is the number of tracts scaled by the sum of inverse of the fiber length and the mean volume of two ROIs (Cheng et al., 2012).

### Statistical analysis

Statistical analyses were conducted using STATA version 13.0 (StataCorp, Colleage Station, Texas, USA) and SigmaPlot v11.0 (Systat Software Inc., Germany). Data were checked for errors and normality assessed for all continuous variables. All data sets were found to be normally distributed.

Paired *t*-tests were used to assess differences between left and right cortical connectivity of SN and between left and right FA values, and unpaired *t*-tests to assess the differences in cortical connectivity between male and female. Reported *P*-values have not been corrected for multiple comparisons, but a *P*-value was considered to be significant only after Bonferroni's correction for multiple comparisons for each of the 33 areas considered (i.e., *P* < 0.0015).

Pearson's correlation was used to test correlations between connectivity and FA for significance. All tests were two-sided.

## Results

In our cohort (*n* = 15), 7 of the subjects were women, the average age was 35.46 ± 11 years.

**Figure 2:**
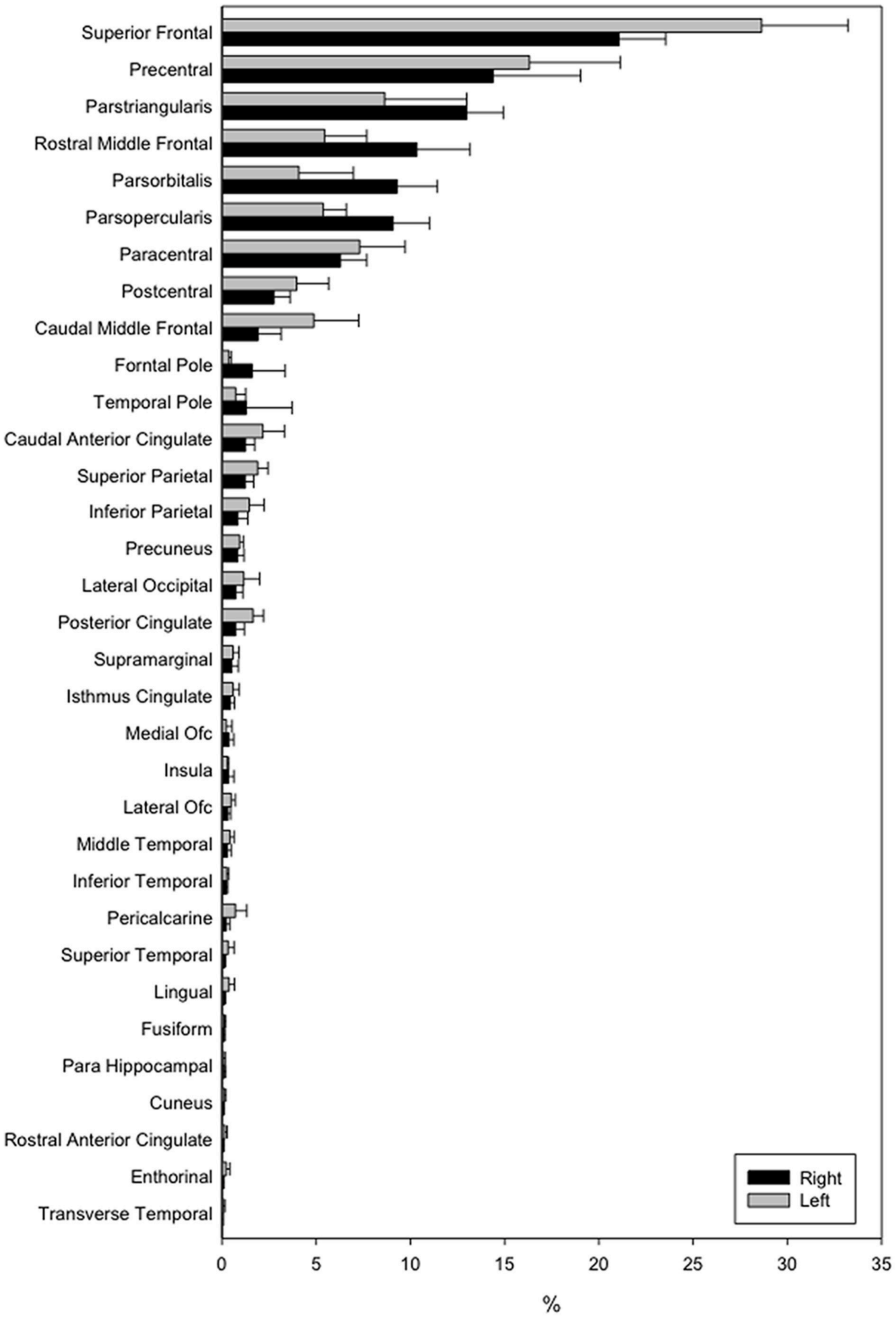
**Connectivity results visualized by means of a bar-graph**.

**Figure 3:**
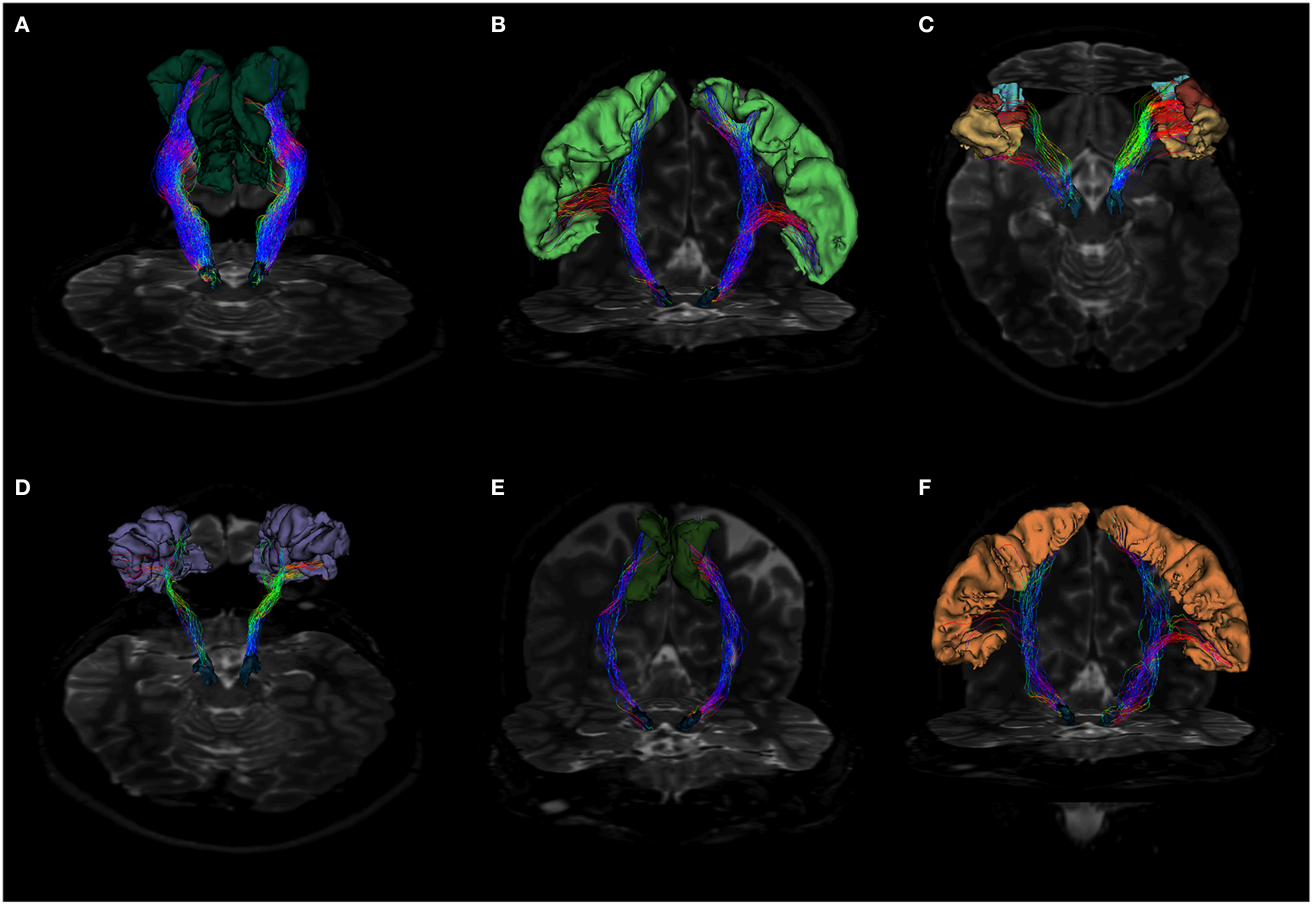
**(A)** Fiber tracts between SN and superior frontal gyrus visualized in a posterior coronal view. SN model is represented in blue, while SFG is colored in dark green. Notice the rich density and organization of these connections. **(B)** Anterior coronal view of fibers between SN and precentral gyrus. SN model is shown in blue, precentral gyrus in pale green. Fiber tracts run through the internal capsule and reach preferentially the posterior and lateral parts of SN. **(C)** Fiber tracts between SN and pars opercularis, pars triangularis and pars orbitalis. SN model is shown in blue, pars opercularis in yellow, pars triangularis in brown and pars orbitalis in light blue. Fibers run through the internal capsule and reach the anterior part of SN. **(D)** Posterior view of fiber tracts between SN and rostral middle frontal gyrus. SN is depicted in blue, while rMFG in pale purple. Streamlines mainly approach the anterior part of SN. **(E)** Fiber tracts between SN and paracentral lobule in anterior coronal view. SN is shown in blue, SMA in green. Fiber tracts mainly interest the posterior part of SN. **(F)** Fiber tracts between SN and postcentral gyrus visualized in anterior coronal view. SN model is depicted in blue, postcentral gyrus in orange. Streamlines spread mainly from the medial regions of postcentral gyrus, but also from the most infero-lateral ones.

Thirty-three consistent patterns of **CSD** tractography-based structural cortical connectivity from the left and right SN were identified (Figure 2). The wast majority of the cortico-nigral connectivity (~83%) was directed toward 8 cortical areas, namely, superior frontal (~25%; Figure 3A), precentral (15.4%; Figure 3B), pars triangularis (10.8%; Figure 3C), rostral middle frontal (7.9%; Figure 3D), pars orbitalis (6.7%; Figure 3C), pars opercularis (7.2%; Figure 3C), paracentral (6.8%; Figure 3E), postcentral (3.6%; Figure 3F; average of the right and left side).

**Figure 4:**
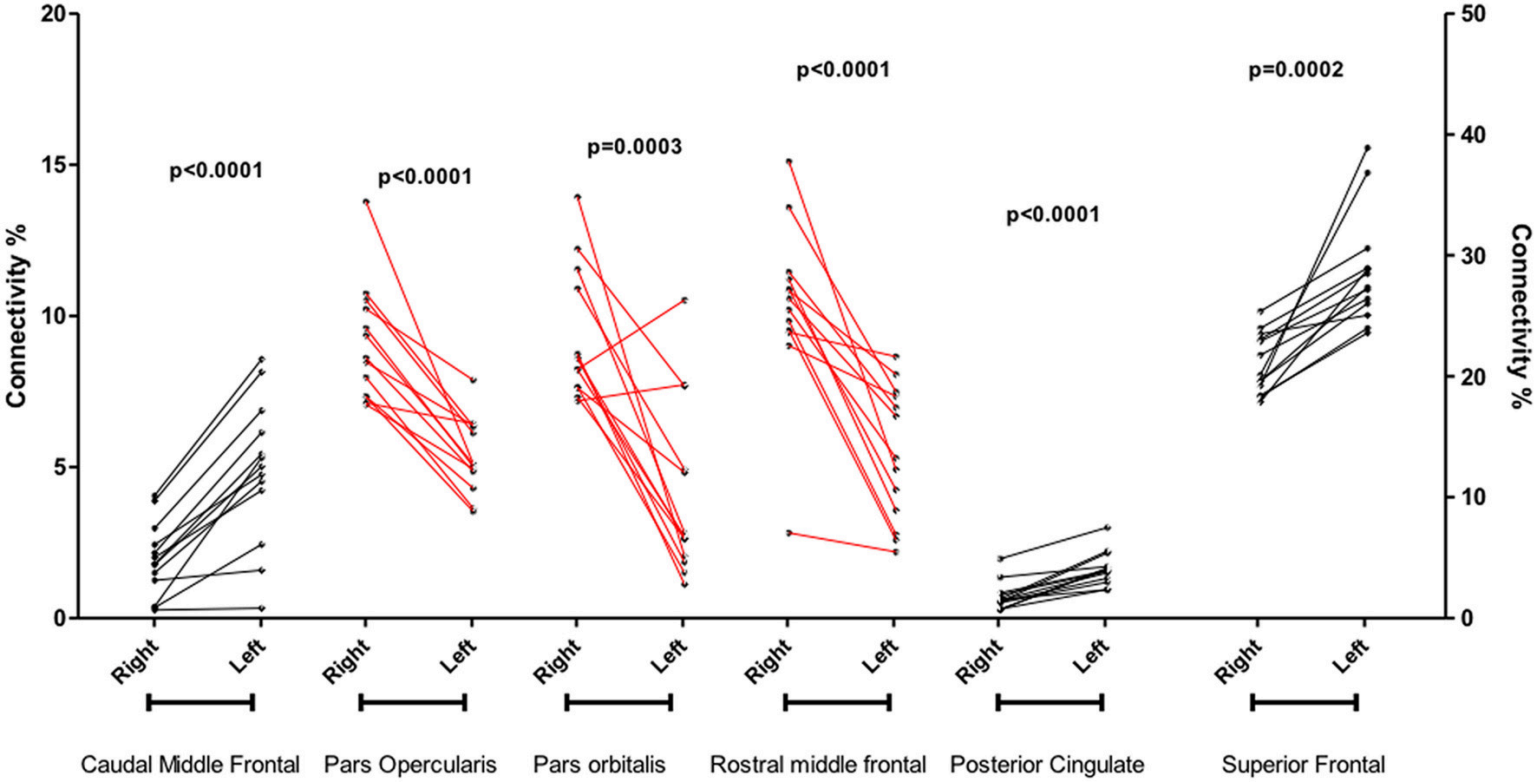
**Significant differences between left and right cortical connectivity of substantia nigra**.

Significant differences were found between left and right cortical connectivity of SN of the caudal middle frontal (*p* < 0.0001), pars opercularis (*p* < 0.0001), posterior cingulate (*p* < 0.0001), rostral middle frontal (*p* < 0.0001), pars orbitalis (*p* = 0.0003), superior frontal (*p* = 0.0002). The pars opercularis, pars orbitalis and rostral middle frontal were predominant right sided, while the others left sided (Figure 4). There was no correlation between connectivity and FA. Connectivity was not associated with gender and did not correlate with age.

**Figure 5:**
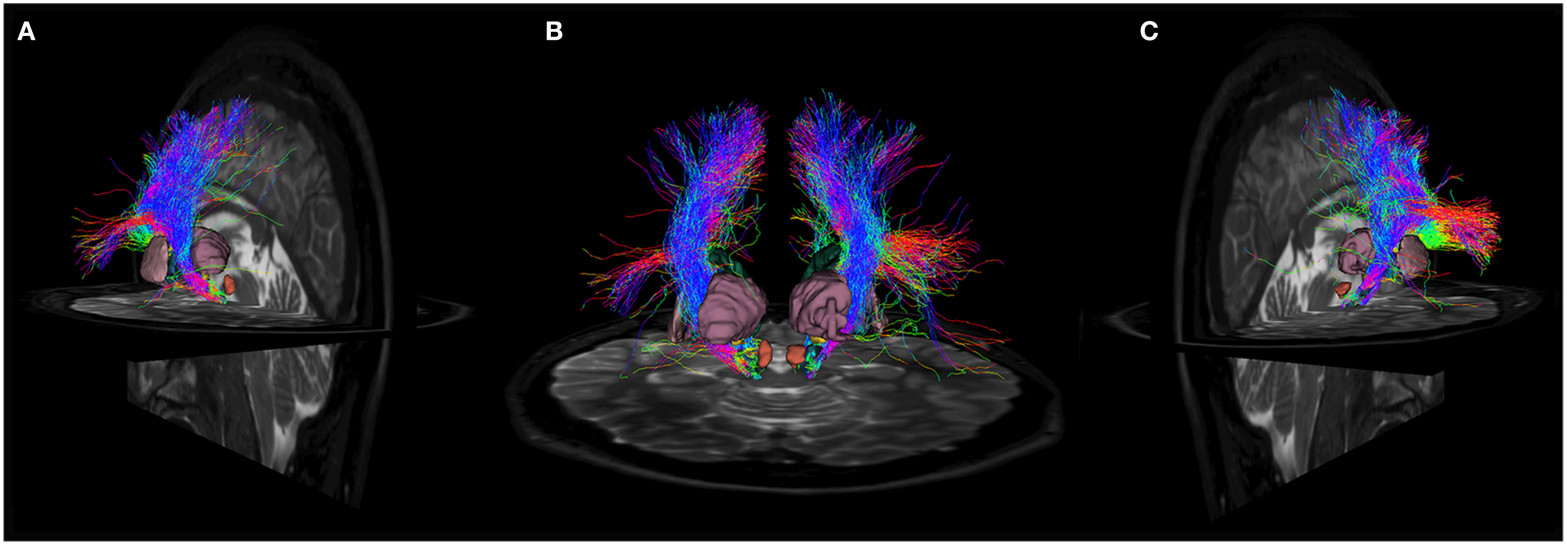
**Anatomical relations of cortico-nigral probabilistic fiber tracking**. In particular, panel **(A)** depicts left cortico-nigral pathway in a posterior parasagittal view; panel **(B)** shows bilateral cortico-nigral tract in posterior coronal view and panel **(C)** shows right cortico-nigral tract in posterior parasagittal view. 3D-rendered models of basal ganglia are shown to better illustrate anatomical relation: SN is shown colored in blue, caudate nucleus in dark green, thalamus in pale purple, putamen in pink, pallidum in light blue, STN in yellow and RN in brown. Cortico-nigral tracts runs mainly through internal capsule, avoiding thalamus, putamen and globus pallidus, and reach SN in the ventral midbrain, bypassing STN and RN.

In particular, the cortico-nigral fibers ran within the internal capsule to the cerebral peduncle, and finally reached the SN in ventral midbrain (Figure 5; Supplementary Figure 1).

Left and right mean FA values are shown in Table 1, whilst Table 2 summarizes our data in comparison to data obtained in animals.

**Table 1:** **Significant differences between left and right mean FA values**.

**FA**	**Right**	**Left**	***P*-value**
Caudal anterior cingulate	0.49 ± 0.041	0.49 ± 0.041	ns
Caudal middle frontal	0.43 ± 0.016	0.43 ± 0.031	ns
Cuneus	0.49 ± 0.031	0.48 ± 0.033	ns
Enthorinal	0.47 ± 0.029	0.47 ± 0.044	ns
Fusiform	0.48 ± 0.023	0.48 ± 0.019	ns
Inferior parietal	0.43 ± 0.024	0.44 ± 0.024	ns
Inferior temporal	0.47 ± 0.022	0.47 ± 0.008	ns
Isthmus cingulate	0.49 ± 0.026	0.49 ± 0.035	ns
Lateral occipital	0.46 ± 0.027	0.45 ± 0.029	ns
Lateral OFC	0.46 ± 0.035	0.47 ± 0.022	ns
Lingual	0.48 ± 0.036	0.47 ± 0.029	ns
Medial OFC	0.48 ± 0.035	0.48 ± 0.023	ns
Middle temporal	0.46 ± 0.017	0.46 ± 0.014	ns
Para hippocampal	0.49 ± 0.028	0.49 ± 0.024	ns
Paracentral	0.42 ± 0.016	0.45 ± 0.023	0.0002
Parsopercularis	0.43 ± 0.018	0.44 ± 0.026	ns
Parsorbitalis	0.42 ± 0.076	0.46 ± 0.023	ns
Parstriangularis	0.42 ± 0.022	0.44 ± 0.034	0.0005
Pericalcarine	0.48 ± 0.043	0.47 ± 0.031	ns
Postcentral	0.42 ± 0.016	0.42 ± 0.021	ns
Posterior cingulate	0.49 ± 0.042	0.49 ± 0.042	ns
Precentral	0.39 ± 0.015	0.4 ± 0.021	ns
Precuneus	0.44 ± 0.023	0.45 ± 0.024	ns
Rostral anterior cingulate	0.5 ± 0.035	0.51 ± 0.032	ns
Rostral middle frontal	0.4 ± 0.016	0.41 ± 0.031	ns
Superior frontal	0.39 ± 0.016	0.4 ± 0.034	ns
Superior parietal	0.42 ± 0.025	0.42 ± 0.02	ns
Superior temporal	0.46 ± 0.018	0.46 ± 0.016	ns
Supramarginal	0.44 ± 0.024	0.45 ± 0.026	ns
Forntal pole	0.48 ± 0.037	0.47 ± 0.023	ns
Temporal pole	0.46 ± 0.032	0.46 ± 0.029	ns
Transverse temporal	0.5 ± 0.034	0.5 ± 0.032	ns
Insula	0.47 ± 0.021	0.47 ± 0.017	ns

*ns, non-significant*.

**Table 2:** **Comparison between our findings in human brain and other results animal brain**.

**Areas involved in rats**	**Areas involved in primates**	**Areas involved in humans**
Medial prefrontal cortex (Sesack et al., 1989) Prefrontal cortex, with sparse fibers to insular, parietal, and temporal cortex (Bunney and Aghajanian, 1976) Motor and premotor areas (Kornhuber, 1984) Orbital area, prelimbic rostral area, anterior cingulate area (Beckstead, 1979; Naito and Kita, 1994) Rostral agranular insular cortex (Jasmin et al., 2004) Primary somatosensory area (Zakiewicz et al., 2014) Olfactory cortex (Höglinger et al., 2015)	Orbital prefrontal cortex, dorso lateral prefrontal cortex, cingulate cortex (Frankle et al., 2006) Area 6 and area 9 (Künzle, 1978; von Monakow et al., 1979)	Superior frontal gyrus Precentral gyrus Pars triangularis Rostral middle frontal gyrus Pars orbitalis Pars opercularis Paracentral gyrus Postcentral gyrus

*For animal findings, we maintained the anatomical nomenclature found in original research articles. References are discussed in text body*.

## Discussion

SN can be anatomically and functionally divided in two sections, the antero-lateral SNr and the postero-medial SNc with significant differences in connectivity profile, receptor-architectonics, and functions (Voorn et al., 2004; Guatteo et al., 2009; Menke et al., 2010; Zhou and Lee, 2011; Chowdhury et al., 2013).

In the present study, we confirmed the existence of an extensive neural pathway running between cerebral cortex and SN, as described by existing literature.

In particular, we noticed the highest connectivity profile for SN with SFG, which is known to be an important prefrontal area; in addiction, lower values of connectivity were found between SN and other prefrontal areas areas such as pars opercularis, pars orbitalis, pars triangularis, and rostral MFG. These findings are in line with data regarding PFC-SN connectivity described in rodents (Bunney and Aghajanian, 1976; Kornhuber, 1984; Sesack et al., 1989) and primates (Leichnetz and Astruc, 1976; Künzle, 1978; Frankle et al., 2006), and with the few existing results obtained in humans by means of DTI-based techniques (Menke et al., 2010; Kwon and Jang, 2014). Interestingly, connections between PFC and SN in humans appears to be more prominent and represented than the sparse dlPFC/SN projections described by Frankle and colleagues in macaca monkeys. On the other hand, we were not able to find representative connections between OFC and SN, in contrast to findings revealed in primates (Frankle et al., 2006).

We also found well-represented connections between SN and cortical areas related with motor functions, such as paracentral lobule and precentral gyrus: this finding is in line with the existing literature that revealed sparse connections between motor cortex, premotor cortex and SN in primates (Künzle, 1978; von Monakow et al., 1979).

In a recent study aimed to examine functional networks related to the extrapyramidal system by using fMRI, it was shown that SN is connected with supplementary motor area (SMA), suggesting the former as an activity driver of the SMA and the latter as a crucial node within the cortico-basal network (Zhang et al., 2015).

Finally, we found significant connections between SN with parietal sensory and sensory-motor cortices. These findings are in keeping with animal data in rodents, obtained with anterograde tracers, that have shown extensive connections running from primary somatosensory area to SNr (Zakiewicz et al., 2014).

This is not the first attempt in demonstrating connections between cerebral cortex and SN in humans. Hence, the existence of direct cortico-nigral fibers has been postulated and demonstrated, back to the first half of XX century, by the French anatomist Leo Testut who, in its classical textbook of anatomy, comments: “Fibers of cerebral cortex, coming from the rolandic region, pass through the internal capsule, with the peduncolar pathway, reach the peduncle's foot and enter the anterior part of SN” (Testut and Latarjet, 1971). A recent study found that SN could be likely connected with prefrontal cortex and sensory-motor cortex, but the authors concluded that these connections were not linking cortex and SN directly, but passing through thalamus (Menke et al., 2010).

By using a probabilistic tractography method based on a multi-fiber model, Kwon and Jang (2014) demonstrated that SN showed high connectivity values to primary motor cortex, primary somatosensory cortex, and premotor cortex and prefrontal cortex. As claimed by the authors, their study suffered from intrinsic technical DTI limitations (Parker and Alexander, 2005; Fillard et al., 2011; Jeurissen et al., 2013) and MRI data were acquired with low Tesla (1.5), channels (6), and diffusion directions (32).

In the present paper, we used a HARDI-CSD-based technique, which is able to provide more robust data, estimating one or more fiber orientations in presence of intravoxel orientational heterogeneity (Tournier et al., 2007, 2008). Moreover, we acquired MRI data with a 3T scanner, 32 channels, *b*-value = 1500 s/mm^2^, and 64 gradient diffusion directions, improving quality of data and reliability of results. Finally, in our work we used exclusion masks for caudate, putamen, thalamus, globus pallidus and STN, and therefore we can assume that this cortico-nigral connectivity is likely to be direct.

Several evidences reinforce the presence of direct cortico-nigral projections in humans. It has been reported that the unilateral ablation of PFC causes a significant reduction of glutamate levels in the ipsilateral SN (Kornhuber, 1984). In addition, there is an old paper suggesting that the pacemaker-like activity of SN neurons can be modulated by afferent cortical projections (Gariano and Groves, 1988).

Finally, there are also indirect evidences suggesting the presence of cortico-nigral connections in humans. Indeed, Strafella and co-workers demonstrated that repetitive transcranial magnetic stimulation over primary motor area and PFC induces dopamine release in caudate nucleus (Strafella et al., 2001).

### Physiopathological implications

The well-known model of basal ganglia physiology put forward by DeLong in the late eighties has plagued the field with a paramount impact on the comprehension of movement disorders (MD), which has boosted neurosurgery for these conditions (DeLong, 1990). The essence of the model postulates that cortical information flows through the basal ganglia via two major systems: the direct and indirect pathways, originating from segregated populations of striatal medium spiny neurons (MNNs) and exerting opposite effects on basal ganglia outflow.

In particular it has been shown that that selective stimulation of the indirect pathway (D1 receptors) provoked movement arrest while activation of direct pathway (D2 receptors) led to movement activation (Bateup et al., 2010).

In addition to the striatum another important node of the network is the STN, which has extensive cortical connections through a fast conductive cortico-subthalamic pathway called hyper-direct pathway (Nambu et al., 2002; Nambu, 2005).

In a recent paper, we have challenged the traditional model of basal ganglia functioning by showing the existence of cortico-pallidal projections in humans (Milardi et al., 2015). By using a CSD-based tractographic approach, we demonstrated the presence of direct pathways connecting Broadmann's areas 4, 5, 6, 11, 12, 46, and 48 with both segments of GP. The cortical output to both segments of GP may provide an efficient system to control upstream direct and indirect pathways. These findings are substantially in line with the emerging view on basal ganglia organization, also supported by several tractographic studies (Draganski et al., 2008; Lambert et al., 2012).

This pathway may provide direct cortical access to GP, bypassing the striatum, and may represent an additional route for cortical regulation of the basal ganglia circuitry (Smith and Wichmann, 2015).

Interestingly, a magnetoencephalography study has recently identified in dystonia patients a robust band of beta coherence between motor and premotor cortical areas with GPi, confirming the functional role of cortico-pallidal beta signaling in regulating the cortex-basal ganglia-cortex feedback loops implicated in motor control (Neumann et al., 2015). We have recently also proposed a new framework where the existence of this abnormal cortico-pallidal connectivity should be explored at a functional and structural level to shed new light on the pathophysiology of movement disorders (Cacciola et al., 2016). In this scenario, we speculated that, as the hyperdirect pathway is a faster connection of cortex with STN, with respect to the direct and indirect pathways, similarly the cortico-pallidal fibers could represent a fast connection between cortex and GP (Milardi et al., 2015; Cacciola et al., 2016).

In the present paper, we provide new evidences supporting the existing model of basal ganglia network physiology by means of tractographic demonstration of a complementary direct cortico-SN pathway that could work paralleling the cortico-pallidal system.

This direct cortico-nigral system is likely to be a very important hierarchically node within the basal ganglia network, as it may exert a powerful upstream control on direct and indirect pathways through a modulation of the nigro-striatal system in addition to the cortico-GPi direct pathway. In addition, we have recently shown a direct connectivity between cerebellum and SN, which may represent a fast route for cerebellar activity to regulate and to be regulated by basal ganglia activity (Milardi et al., 2016a). In this perspective, cortico-nigral-cerebellar connections may directly interact each other resulting in a fine regulation of SNr firing.

However, based on MRI data used in the present study, we cannot discriminate between the two different portions of SN.

SNr is, along with internal globus pallidus (GPi), the output nucleus in basal ganglia circuitry, since it fires directly on VA and VL thalamic nuclei with inhibitory GABA-ergic neurons. The regular high-frequency spontaneous firing of SNr neurons is mainly modulated by inhibitory neurons from striatum and external globus pallidus (GPe) and excitatory neurons from STN (Zhou and Lee, 2011).

The results of the present study are very relevant in light of recent findings that have demonstrated an important role of SNr in the pathophysiology of hyperkinetic MD providing an explanation of the paradox of pallidotomy in PD. It is well-known that, according to the traditional model of DeLong, a lesion of the GP should reduce GPi efferent activity worsening the dyskinesias (DeLong, 1990). However, clinical data in several hundreds of treated patients suggest the opposite (Bejjani et al., 1997; Guridi and Lozano, 1997; Lozano and Lang, 2001; Wu et al., 2001; Volkmann et al., 2002; Munhoz et al., 2014).

A partial solution of this paradox has been provided by Dybdal and colleagues who have demonstrated that experimental inhibition of a very restricted area of SNr can induce a choreiform dyskinesia which is distinct from that responsible for inducing torticollis (Dybdal et al., 2013).

The contralateral directed choreiform dyskinesias and torticollis observed after focal inhibition within specific regions of the SNr can explain some of the symptoms observed in several MD such as, Huntington's disease, levodopa-induced dyskinesias, cranio-cevical and generalized dystonias, and tardive dyskinesias and dystonias.

The crucial role of SNr in MD can also explain why the excessive activation of glutamatergic subthalamo-SNr and GABAergic pallido-SNr fibers, driven by STN DBS may produce dyskinesias (Bosch et al., 2011).

Strategies aimed at disinhibition of the SNr warrant further exploration as potential therapeutic interventions for human torticollis and dyskinetic syndromes. In this perspective, cortical modulation with non-invasive neuromodulation technique could offer an alternative opportunity to modulate SN firing.

## Limitations

The present study regarding cerebral-SN connectivity suffers from some limitations. It is worthy to note that tractography does not provide a direct visualization of axons, allowing only a probabilistic representation of the most likely trajectories based on local diffusion.

A drawback of this technique is also the inability to establish the directionality of signal transmission (Chung et al., 2011; Parker et al., 2013) that need to be determined with functional connectivity approaches. The latter could use technique such as dynamic causal modeling to infer whether firing of one brain region leads to activity changes in other regions, thus determining directionality of these connections. A combined approach between dynamic causal modeling-based functional studies together with the tracking method employed in the present study, should be fostered to resolve issue directionality of this cortico-basal ganglia pathway. Moreover, tractography cannot provide fully conclusive evidence of direct anatomical connections since it is unable to disentangle monosynaptic from polysynaptic connections and to detect gap junctions. Further studies need to be carried out in the human brain using postmortem microsurgery dissection and/or tracer injection or *in vivo* fMRI in order to confirm the existence of this cortico-nigral pathway.

A further limitation of the present study is that we could not discriminate between cortical direct connectivity of SNc and SNr, since images resolution didn't allow for it. However, this could be the aim of further investigations, by using higher resolution acquisition and 7T scanners.

Although our results are anatomically plausible compared with previous anatomical descriptions of these connections in animals, tractography results have to be interpreted with caution. Hence, it is well-known that tractography may suffer from reconstruction biases, such as possible false positive streamlines. In this regard, to make our tractographic findings more reliable we decided to use probabilistic CSD tractography with more conservative reconstruction criteria respecting usual standard (Descoteaux et al., 2009; Milardi et al., 2015), at the cost of an underestimation issue.

Finally, our results are based on a relatively small sample of adult subjects, and this might explain why we have not found any correlations between sex and age.

However, despite its limitations, it is worthy to remember that tractography is the only available technique to investigate structural neural connectivity *in vivo* and non-invasively.

## Conclusions

Using diffusion-tensor magnetic resonance imaging and CSD tractography, we demonstrated the presence of direct anatomical cortico-nigral connections, already extensively described in cats, rodents and primates but only suggested in humans.

This direct connection might be another piece of the puzzle toward a more extensive comprehension of cortical regulation on both motor basal ganglia and dopaminergic reward circuits and might pave the way for further biochemical and physiological investigations.

## Author contributions

AC: Study concepts/study design, data acquisition, data analysis, data interpretation, literature research. DM: Study concepts/study design, manuscript revision. GA: Guarantor of integrity of entire study, approval of final version of submitted manuscript, data interpretation. GB: Data analysis, data interpretation, literature research, statistical analysis. MI: Data analysis, literature research, statistical analysis. PC: Data analysis, data interpretation. GC: Literature research. DB: Literature research. GR: Data acquisition. SM: Statistical analysis, data analysis. PB: Manuscript revision. AQ: Study concepts/study design, guarantor of integrity of entire study, manuscript revision for important intellectual content.

### Conflict of interest statement

The authors declare that the research was conducted in the absence of any commercial or financial relationships that could be construed as a potential conflict of interest.

## Supplementary material

Supplementary Figure 1 **Course of cortico-nigral connections**. Representation of the course of the connections between cerebral cortex and SN. On the right fibers are red colored, on the left blue colored. The first two images depict tracts leaving the cerebral cortex, then fibers pass through corona radiata and internal capsule to finally reach SN.

## References

[B1] Afifi A. K. Bahuth N. B. Kaelber W. W. Mikhael E. Nassar S. (1974). The cortico-nigral fibre tract.An experimental Fink-Heimer study in cats. J. Anat. 118, 469–476. 4141704 PMC1231545

[B2] Alexander D. C. Barker G. J. (2005). Optimal imaging parameters for fiber-orientation estimation in diffusion MRI. Neuroimage 27, 357–367. 10.1016/j.neuroimage.2005.04.008 15921931

[B3] Basser P. J. (1995). Inferring microstructural features and the physiological state of tissues from diffusion-weighted images. NMR Biomed. 8, 333–344. 10.1002/nbm.1940080707 8739270

[B4] Basser P. J. Mattiello J. LeBihan D. (1994). MR diffusion tensor spectroscopy and imaging. Biophys. J. 66, 259–267. 10.1016/S0006-3495(94)80775-1 8130344 PMC1275686

[B5] Bateup H. S. Santini E. Shen W. Birnbaum S. Valjent E. Surmeier D. J.. (2010). Distinct subclasses of medium spiny neurons differentially regulate striatal motor behaviors. Proc. Natl. Acad. Sci. U.S.A. 107, 14845–14850. 10.1073/pnas.1009874107 20682746 PMC2930415

[B6] Beckstead R. M. (1979). An autoradiographic examination of corticocortical and subcortical projections of the mediodorsal-projection (prefrontal) cortex in the rat. J. Comp. Neurol. 184, 43–62. 10.1002/cne.901840104 762282

[B7] Behrens T. E. Berg H. J. Jbabdi S. Rushworth M. F. Woolrich M. W. (2007). Probabilistic diffusion tractography with multiple fibre orientations: what can we gain? Neuroimage 34, 144–155. 10.1016/j.neuroimage.2006.09.018 17070705 PMC7116582

[B8] Behrens T. E. Sporns O. (2012). Human connectomics. Curr. Opin. Neurobiol. 22, 144–153. 10.1016/j.conb.2011.08.005 21908183 PMC3294015

[B9] Bejjani B. Damier P. Arnulf I. Bonnet A. M. Vidailhet M. Dormont D.. (1997). Pallidal stimulation for Parkinson's disease. Two targets? Neurology 49, 1564–1569. 10.1212/WNL.49.6.1564 9409347

[B10] Besson P. Dinkelacker V. Valabregue R. Thivard L. Leclerc X. Baulac M.. (2014). Structural connectivity differences in left and right temporal lobe epilepsy. Neuroimage 100, 135–144. 10.1016/j.neuroimage.2014.04.071 24814212

[B11] Bijttebier S. Caeyenberghs K. van den Ameele H. Achten E. Rujescu D. Titeca K.. (2015). The vulnerability to suicidal behavior is associated with reduced connectivity strength. Front. Hum. Neurosci. 9:632. 10.3389/fnhum.2015.00632 26648857 PMC4663245

[B12] Bosch C. Degos B. Deniau J. M. Venance L. (2011). Subthalamic nucleus high-frequency stimulation generates a concomitant synaptic excitation-inhibition in substantia nigra pars reticulata. J. Physiol. 589(Pt 17), 4189–4207. 10.1113/jphysiol.2011.211367 21690190 PMC3180578

[B13] Bunney B. S. Aghajanian G. K. (1976). The precise localization of nigral afferents in the rat as determined by a retrograde tracing technique. Brain Res. 117, 423–435. 10.1016/0006-8993(76)90751-4 990939

[B14] Cacciola A. Milardi D. Quartarone A. (2016). Role of cortico-pallidal connectivity in the pathophysiology of dystonia. Brain.. [Epub ahead of print]. 10.1093/brain/aww102 27190024

[B15] Carman J. B. (1968). Anatomic basis of surgical treatment of Parkinson's disease. N. Engl. J. M. 17, 919–930. 10.1056/NEJM196810242791706 20617599

[B16] Carter C. J. (1982). Topographical distribution of possible glutamatergic pathways from the frontal cortex to the striatum and substantia nigra in rats. Neuropharmacology 21, 379–383. 10.1016/0028-3908(82)90019-3 7110527

[B17] Cheng H. Wang Y. Sheng J. Kronenberger W. G. Mathews V. P. Hummer T. A.. (2012). Characteristics and variability of structural networks derived from diffusion tensor imaging. Neuroimage 61, 1153–1164. 10.1016/j.neuroimage.2012.03.036 22450298 PMC3500617

[B18] Chowdhury R. Lambert C. Dolan R. J. Düzel E. (2013). Parcellation of the human substantia nigra based on anatomical connectivity to the striatum. Neuroimage 81, 191–198. 10.1016/j.neuroimage.2013.05.043 23684858 PMC3734352

[B19] Chung H. W. Chou M. C. Chen C. Y. (2011). Principles and limitations of computational algorithms in clinical diffusion tensor MR tractography. Am. J. Neuroradiol. 32, 3–13. 10.3174/ajnr.A2041 20299436 PMC7964942

[B20] DeLong M. R. (1990). Primate models of movement disorders of basal ganglia origin. Trends. Neurosci. 13, 281–285. 10.1016/0166-2236(90)90110-V 1695404

[B21] Descoteaux M. Deriche R. Knösche T. R. Anwander A. (2009). Deterministic and probabilistic tractography based on complex fibre orientation distributions. IEEE Trans. Med. Imaging. 28, 269–286. 10.1109/TMI.2008.2004424 19188114

[B22] Desikan R. S. Ségonne F. Fischl B. Quinn B. T. Dickerson B. C. Blacker D.. (2006). An automated labeling system for subdividing the human cerebral cortex on MRI scans into gyral based regions of interest. Neuroimage 31, 968–980. 10.1016/j.neuroimage.2006.01.021 16530430

[B23] Draganski B. Kherif F. Klöppel S. Cook P. A. Alexander D. C. Parker G. J.. (2008). Evidence for segregated and integrative connectivity patterns in the human basal ganglia. J. Neurosci. 28, 7143–7152. 10.1523/JNEUROSCI.1486-08.2008 18614684 PMC6670486

[B24] Düzel E. Bunzeck N. Guitart-Masip M. Wittmann B. Schott B. H. Tobler P. N. (2009). Functional imaging of the human dopaminergic midbrain. Trends Neurosci. 32, 321–328. 10.1016/j.tins.2009.02.005 19446348

[B25] Dybdal D. Forcelli P. A. Dubach M. Oppedisano M. Holmes A. Malkova L.. (2013). Topography of dyskinesias and torticollis evoked by inhibition of substantia nigra pars reticulate. Mov. Disord. 28, 460–468. 10.1002/mds.25215 23115112

[B26] Embleton K. V. Haroon H. A. Morris D. M. Ralph M. A. Parker G. J. (2010). Distortion correction for diffusion-weighted MRI tractography and fMRI in the temporal lobes. Hum. Brain Mapp. 31, 1570–1587. 10.1002/hbm.20959 20143387 PMC6870737

[B27] Farquharson S. Tournier J. D. Calamante F. Fabinyi G. Schneider-Kolsky M. Jackson G. D.. (2013). White matter fiber tractography: why we need to move beyond DTI. J. Neurosurg. 118, 1367–1377. 10.3171/2013.2.JNS121294 23540269

[B28] Fillard P. Descoteaux M. Goh A. Gouttard S. Jeurissen B. Malcolm J.. (2011). Quantitative evaluation of 10 tractography algorithms on a realistic diffusion MR phantom. Neuroimage 56, 220–234. 10.1016/j.neuroimage.2011.01.032 21256221

[B29] Fischl B. Dale A. M. (2000). Measuring the thickness of the human cerebral cortex from magnetic resonance images. Proc. Natl. Acad. Sci. U.S.A. 97, 11050–11055. 10.1073/pnas.200033797 10984517 PMC27146

[B30] Fischl B. Salat D. H. van der Kouwe A. J. Makris N. Ségonne F. Quinn B. T.. (2004). Sequence-independent segmentation of magnetic resonance images. Neuroimage 23(Suppl. 1), S69–S84. 10.1016/j.neuroimage.2004.07.016 15501102

[B31] Foix C. Niculescu I. T. (1925). Anatomie Cérébrale; Les Noyaux Gris Centraux et la Région Mésencéphalo-Sous-Optique, Suivi d'un Appendice sur L'anatomie Pathologique de la Maladie de Parkinson. Paris: Masson et cie.

[B32] Frankle W. G. Laruelle M. Haber S. N. (2006). Prefrontal cortical projections to the midbrain in primates: evidence for a sparse connection. Neuropsychopharmacology 31, 1627–1636. 10.1038/sj.npp.1300990 16395309

[B33] Gariano R. F. Groves P. M. (1988). Burst firing induced in midbrain dopamine neurons by stimulation of the medial prefrontal and anterior cingulate cortices. Brain Res. 462, 194–198. 10.1016/0006-8993(88)90606-3 3179734

[B34] Grèzes J. Valabrègue R. Gholipour B. Chevallier C. (2014). A direct amygdala-motor pathway for emotional displays to influence action: a diffusion tensor imaging study. Hum. Brain Mapp. 35, 5974–5983. 10.1002/hbm.22598 25053375 PMC6869045

[B35] Guatteo E. Cucchiaroni M. L. Mercuri N. B. (2009). Substantia nigra control of basal ganglia nuclei. J. Neural Transm. Suppl. 73, 91–101. 10.1007/978-3-211-92660-4_7 20411770

[B36] Guridi J. Lozano A. M. (1997). A brief history of pallidotomy. Neurosurgery 41, 1169–1180. 10.1097/00006123-199711000-00029 9361073

[B37] Henderson J. M. (2012). “Connectomic surgery”: diffusion tensor imaging (DTI) tractography as a targeting modality for surgical modulation of neural networks. Front. Integr. Neurosci. 6:15. 10.3389/fnint.2012.00015 22536176 PMC3334531

[B38] Höglinger G. U. Alvarez-Fischer D. Arias-Carrión O. Djufri M. Windolph A. Keber U.. (2015). A new dopaminergic nigro-olfactory projection. Acta Neuropathol. 130, 333–348. 10.1007/s00401-015-1451-y 26072303

[B39] Jang S. H. Son S. M. Lee D. Y. Hong J. H. (2013). Relationship between somatosensory function and the spinothalamocortical pathway in chronic stroke patients. Somatosens. Mot. Res. 30, 197–200. 10.3109/08990220.2013.790808 23697637

[B40] Jasmin L. Granato A. Ohara P. T. (2004). Rostral agranular insular cortex and pain areas of the central nervous system: a tract-tracing study in the rat. J. Comp.Neurol. 468, 425–440. 10.1002/cne.10978 14681935

[B41] Jbabdi S. Johansen-Berg H. (2011). Tractography: where do we go from here? Brain Connect. 1, 169–183. 10.1089/brain.2011.0033 22433046 PMC3677805

[B42] Jbabdi S. Sotiropoulos S. N. Haber S. N. Van Essen D. C. Behrens T. E. (2015). Measuring macroscopic brain connections *in vivo*. Nat. Neurosci. 18, 1546–1555. 10.1038/nn.4134 26505566

[B43] Jeurissen B. Leemans A. Tournier J. D. Jones D. K. Sijbers J. (2013). Investigating the prevalence of complex fiber configurations in white matter tissue with diffusion magnetic resonance imaging. Hum. Brain Mapp. 34, 2747–2766. 10.1002/hbm.22099 22611035 PMC6870534

[B44] Jones D. K. Basser P. J. (2004). “Squashing peanuts and smashing pumpkins”: how noise distorts diffusion-weighted MR data. Magn. Reson. Med. 52, 979–993. 10.1002/mrm.20283 15508154

[B45] Jones D. K. Cercignani M. (2010). Twenty-five pitfalls in the analysis of diffusion MRI data. NMR Biomed. 23, 803–820. 10.1002/nbm.1543 20886566

[B46] Kita H. (2001). Neostriatal and globus pallidus stimulation induced inhibitory postsynaptic potentials in entopeduncular neurons in rat brain slice preparations. Neuroscience 105, 871–879. 10.1016/S0306-4522(01)00231-7 11530225

[B47] Kornhuber J. (1984). The cortico-nigral projection: reduced glutamate content in the substantia nigra following frontal cortex ablation in the rat. Brain Res. 322, 124–126. 10.1016/0006-8993(84)91189-2 6151416

[B48] Künzle H. (1978). An autoradiographic analysis of the efferent connections from premotor and adjacent prefrontal regions (areas 6 and 9) in macaca fascicularis. Brain Behav. Evol. 15, 185–234. 10.1159/000123779 99205

[B49] Kwon H. G. Jang S. H. (2014). Differences in neural connectivity between the substantia nigra and ventral tegmental area in the human brain. Front. Hum. Neurosci. 8:41. 10.3389/fnhum.2014.00041 24567711 PMC3915097

[B50] Lambert C. Zrinzo L. Nagy Z. Lutti A. Hariz M. Foltynie T.. (2012). Confirmation of functional zones within the human subthalamic nucleus: patterns of connectivity and sub-parcellation using diffusion weighted imaging. Neuroimage 60, 83–94 10.1016/j.neuroimage.2011.11.082 22173294 PMC3315017

[B51] Le Bihan D. Johansen-Berg H. (2012). Diffusion MRI at 25: exploring brain tissue structure and function. Neuroimage 61, 324–341 10.1016/j.neuroimage.2011.11.006 22120012 PMC3683822

[B52] Leblois A. Boraud T. Meissner W. Bergman H. Hansel D. (2006). Competition between feedback loops underlies normal and pathological dynamics in the basal ganglia. J. Neurosci. 26, 3567–3583. 10.1523/JNEUROSCI.5050-05.2006 16571765 PMC6673853

[B53] Leichnetz G. R. Astruc J. (1976). The efferent projections of the medial prefrontal cortex in the squirrel monkey (*Saimiri sciureus*). Brain Res. 109, 455–472. 10.1016/0006-8993(76)90027-5 819102

[B54] Li C. Huang B. Zhang R. Ma Q. Yang W. Wang L. (2016). Impaired topological architecture of brain structural networks in idiopathic Parkinson's disease: a DTI study. Brain Imaging Behav.. [Epub ahead of print]. 10.1007/s11682-015-9501-6 26815739

[B55] Lozano A. M. Lang A. E. (2001). Pallidotomy for Parkinson's disease. Adv. Neurol. 86, 413–420. 11554004

[B56] McFarland N. R. Haber S. N. (2000). Convergent inputs from thalamic motor nuclei and frontal cortical areas to the dorsal striatum in the primate. J. Neurosci. 20, 3798–3813. 10804220 10.1523/JNEUROSCI.20-10-03798.2000 PMC6772665

[B57] Menke R. A. Jbabdi S. Miller K. L. Matthews P. M. Zarei M. (2010). Connectivity-based segmentation of the substantia nigra in human and its implications in Parkinson's disease. Neuroimage 52, 1175–1180. 10.1016/j.neuroimage.2010.05.086 20677376

[B58] Milardi D. Arrigo A. Anastasi G. Cacciola A. Marino S. Mormina E.. (2016a). Extensive direct subcortical cerebellum-basal ganglia connections in human brain as revealed by constrained spherical deconvolution tractography. Front. Neuroanat. 10:29. 10.3389/fnana.2016.00029 27047348 PMC4796021

[B59] Milardi D. Cacciola A. Cutroneo G. Marino S. Irrera M. Cacciola G.. (2016b). Red nucleus connectivity as revealed by constrained spherical deconvolution tractography. Neurosci. Lett. 626, 68–73. 10.1016/j.neulet.2016.05.009 27181514

[B60] Milardi D. Gaeta M. Marino S. Arrigo A. Vaccarino G. Mormina E.. (2015). Basal ganglia network by constrained spherical deconvolution: a possible cortico-pallidal pathway? Mov. Disord. 30, 342–349. 10.1002/mds.25995 25156805

[B61] Mori S. Wakana S. Van Zijl P. C. M. (2004). MRI Atlas of Human White Matter. Amsterdam, San Diego, CA: Elsevier.

[B62] Mormina E. Arrigo A. Calamuneri A. Granata F. Quartarone A. Ghilardi M. F.. (2015). Diffusion tensor imaging parameters' changes of cerebellar hemispheres in Parkinson's disease. Neuroradiology 57, 327–334. 10.1007/s00234-014-1473-5 25479963

[B63] Munhoz R. P. Cerasa A. Okun M. S. (2014). Surgical treatment of dyskinesia in Parkinson's disease. Front. Neurol. 5:65. 10.3389/fneur.2014.00065 24808889 PMC4010755

[B64] Naito A. Kita H. (1994). The cortico-nigral projection in the rat: an anterograde tracing study with biotinylated dextran amine. Brain Res. 637, 317–322. 10.1016/0006-8993(94)91252-1 7514084

[B65] Nambu A. (2005). A new approach to understand the pathophysiology of Parkinson's disease. J. Neurol. 252(Suppl. 4), IV1–IV4. 10.1007/s00415-005-4002-y 16222431

[B66] Nambu A. Takada M. Inase M. Tokuno H. (1996). Dual somatotopical representations in the primate subthalamic nucleus: evidence for ordered but reversed body-map transformations from the primary motor cortex and the supplementary motor area. J. Neurosci. 16, 2671–2683. 8786443 10.1523/JNEUROSCI.16-08-02671.1996 PMC6578767

[B67] Nambu A. Tokuno H. Hamada I. Kita H. Imanishi M. Akazawa T.. (2000). Excitatory cortical inputs to pallidal neurons via the subthalamic nucleus in the monkey. J. Neurophysiol. 84, 289–300. 10899204 10.1152/jn.2000.84.1.289

[B68] Nambu A. Tokuno H. Takada M. (2002). Functional significance of the cortico-subthalamo-pallidal ‘hyperdirect’ pathway. Neurosci. Res. 43, 111–117. 10.1016/S0168-0102(02)00027-5 12067746

[B69] Nestler E. J. Hyman S. E. Malenka R. C. (2009). Molecular Neuropharmacology: A Foundation for Clinical Neuroscience. New York, NY: McGraw-Hill Medical.

[B70] Neumann W. J. Jha A. Bock A. Huebl J. Horn A. Schneider G. H.. (2015). Cortico-pallidal oscillatory connectivity in patients with dystonia. Brain 138, 1894–1906. 10.1093/brain/awv109 25935723

[B71] Pajevic S. Pierpaoli C. (1999). Colour schemes to represent the orientation of anisotropic tissues from diffusion tensor data: application to white matter fiber tract mapping in the human brain. Magn. Reson. Med. 42, 526–540. 10467297

[B72] Parker G. D. Marshall D. Rosin P. L. Drage N. Richmond S. Jones D. K. (2013). A pitfall in the reconstruction of fibre ODfs using spherical deconvolution of diffusion MRI data. Neuroimage 65, 433–448. 10.1016/j.neuroimage.2012.10.022 23085109 PMC3580290

[B73] Parker G. J. Alexander D. C. (2005). Probabilistic anatomical connectivity derived from the microscopic persistent angular structure of cerebral tissue. Philos. Trans. R. Soc. Lond. B. Biol. Sci. 360, 893–902. 10.1098/rstb.2005.1639 16087434 PMC1854923

[B74] Reuter M. Rosas H. D. Fischl B. (2010). Highly accurate inverse consistent registration: a robust approach. Neuroimage 53, 1181–1196. 10.1016/j.neuroimage.2010.07.020 20637289 PMC2946852

[B75] Rinvik E. (1966). The cortico-nigral projection in the cat. Brain Res. 126, 241–254. 5935375 10.1002/cne.901260206

[B76] Sakai S. T. (1978). Corticonigral projections from area 6 in the raccoon. Exp. Brain Res. 73, 498–504. 10.1007/BF00406607 3224659

[B77] Segonne F. Dale A. M. Busa E. Glessner M. Salat D. Hahn H. K.. (2004). A hybrid approach to the skull stripping problem in MRI. Neuroimage 22, 1060–1075. 10.1016/j.neuroimage.2004.03.032 15219578

[B78] Ségonne F. Pacheco J. Fischl B. (2007). Geometrically accurate topology-correction of cortical surfaces using nonseparating loops. IEEE Trans. Med. Imaging 26, 518–529. 10.1109/TMI.2006.887364 17427739

[B79] Sesack S. R. Deutch A. Y. Roth R. H. Bunney B. S. (1989). Topographical organization of the efferent projections of the medial prefrontal cortex in the rat: an anterograde tract-tracing study with Phaseolus vulgaris leucoagglutinin. J. Comp. Neurol. 290, 213–242. 10.1002/cne.902900205 2592611

[B80] Smith R. E. Tournier J. D. Calamante F. Connelly A. (2013). SIFT: spherical-deconvolution informed filtering of tractograms. Neuroimage 67, 298–312. 10.1016/j.neuroimage.2012.11.049 23238430

[B81] Smith Y. Wichmann T. (2015). The cortico-pallidal projection: an additional route for cortical regulation of the basal ganglia circuitry. Mov. Disord. M 30, 293–295. 10.1002/mds.26095 25476969 PMC4357539

[B82] Strafella A. P. Paus T. Barrett J. Dagher A. (2001). Repetitive transcranial magnetic stimulation of the human prefrontal cortex induces dopamine release in the caudate nucleus. J. Neurosci. 21, RC157. 11459878 10.1523/JNEUROSCI.21-15-j0003.2001 PMC6762641

[B83] Testut L. Latarjet A. (1971). Anatomia Umana. Vol. 3 Torino: UTET.

[B84] Tournier J. D. Calamante F. Connelly A. (2007). Robust determination of the fibre orientation distribution in diffusion MRI: non-negativity constrained super-resolved spherical deconvolution. Neuroimage 35, 1459–1472. 10.1016/j.neuroimage.2007.02.016 17379540

[B85] Tournier J. D. Calamante F. Connelly A. (2011). Effect of step size on probabilistic streamlines: implications for the interpretation of connectivity analysis. Proc. Intl. Soc. Mag Reson. Med. 19, 2019.

[B86] Tournier J. D. Yeh C. H. Calamante F. Cho K. H. Connelly A. Lin C. P. (2008). Resolving crossing fibres using constrained spherical deconvolution: validation using diffusion-weighted imaging phantom data. Neuroimage 42, 617–625. 10.1016/j.neuroimage.2008.05.002 18583153

[B87] Verstynen T. Jarbo K. Pathak S. Schneider W. (2011). *In vivo* mapping of microstructural somatotopies in the human corticospinal pathways. J. Neurophysiol. 105, 336–346. 10.1152/jn.00698.2010 21068263

[B88] Volkmann J. Herzog J. Kopper F. Deuschl G. (2002). Introduction to the programming of deep brain stimulators. Mov. Disord. 17, S181–S187. 10.1002/mds.10162 11948775

[B89] von Monakow K. H. Akert K. Künzle H. (1979). Projections of precentral and premotor cortex to the red nucleus and other midbrain areas in Macaca fascicularis. Exp. Brain Res. 34, 91–105. 10.1007/BF00238343 83242

[B90] Voorn P. Vanderschuren L. J. Groenewegen H. J. Robbins T. W. Pennartz C. M. (2004). Putting a spin on the dorsal-ventral divide of the striatum. Trends Neurosci. 27, 468–474. 10.1016/j.tins.2004.06.006 15271494

[B91] Weinberger D. R. (1987). Implications of the normal brain development for the pathogenesis of schizophrenia. Arch. Gen. Psychiatry 44, 660–669. 10.1001/archpsyc.1987.01800190080012 3606332

[B92] Wise R. A. (2009). Roles for nigrostriatal–not just mesocorticolimbic–dopamine in reward and addiction. Trends Neurosci. 32, 517–524. 10.1016/j.tins.2009.06.004 19758714 PMC2755633

[B93] Wu Y. R. Levy R. Ashby P. Tasker R. R. Dostrovsky J. O. (2001). Does stimulation of the GPi control dyskinesia by activating inhibitory axons? Mov. Disord. 16, 208–216. 10.1002/mds.1046 11295772

[B94] Zakiewicz I. M. Bjaalie J. G. Leergaard T. B. (2014). Brain-wide map of efferent projections from rat barrel cortex. Front. Neuroinform. 8:5. 10.3389/fninf.2014.00005 24550819 PMC3914153

[B95] Zhang H. Y. Tang H. Chen W. X. Ji G. J. Ye J. Wang N.. (2015). Mapping the functional connectivity of the substantia nigra, red nucleus and dentate nucleus: a network analysis hypothesis associated with the extrapyramidal system. Neurosci. Lett. 606, 36–41. 10.1016/j.neulet.2015.08.029 26342496

[B96] Zhou F. M. Lee C. R. (2011). Intrinsic and integrative properties of substantia nigra pars reticulata neurons. Neuroscience 198, 69–94. 10.1016/j.neuroscience.2011.07.061 21839148 PMC3221915

