# Supplementary figures and images for: A Direct Cortico-Nigral Pathway as Revealed by Constrained Spherical Deconvolution Tractography in Humans
Source: Front Hum Neurosci. 2016 Jul 26;10:374. doi: 10.3389/fnhum.2016.00374 (PMC4960230; doi:10.3389/fnhum.2016.00374)

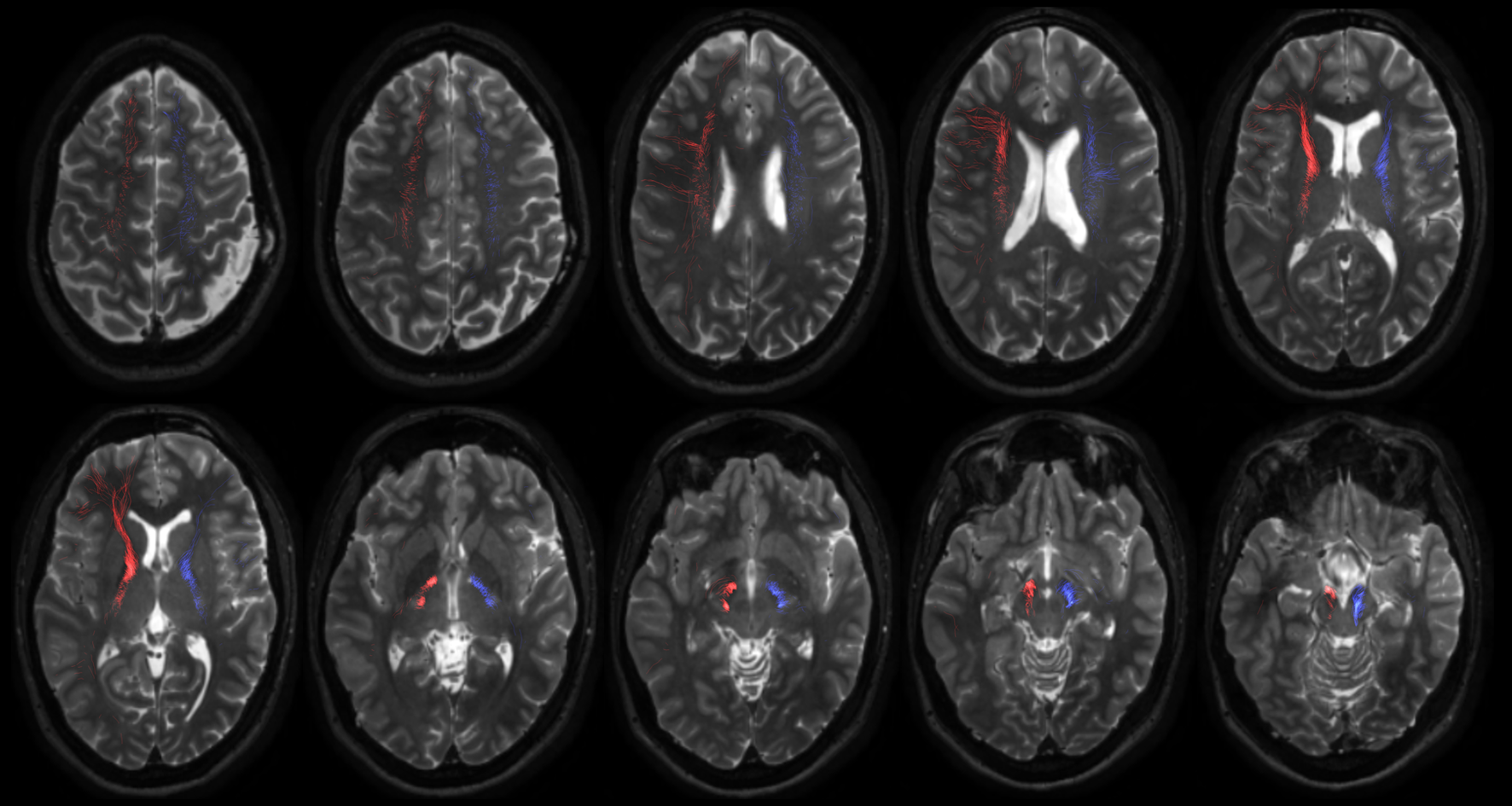

Supplement: Supplementary Figure 1 — Course of cortico-nigral connections. Representation of the course of the connections between cerebral cortex and SN. On the right fibers are red colored, on the left blue colored. The first two images depict tracts leaving the cerebral cortex, then fibers pass through corona radiata and internal capsule to finally reach SN. [file Image1.TIF]
